# Primary hepatic malignant vascular tumors: a follow-up study of imaging characteristics and clinicopathological features

**DOI:** 10.1186/s40644-020-00336-9

**Published:** 2020-08-14

**Authors:** Yue Zhou, Ping Hou, Feng Wang, Bingjie Li, Jianbo Gao

**Affiliations:** 1 Department of Radiology, The First Affiliated Hospital of Zhengzhou University, No. 1 East Jianshe Road, Zhengzhou, Henan China; 2 Department of Oncology, The First Affiliated Hospital of Zhengzhou University, Zhengzhou, Henan China

**Keywords:** Liver neoplasms, Angiosarcoma, Epithelioid hemangioendothelioma, Hemangiopericytoma, CT, MRI, Pathology

## Abstract

**Background:**

Owing to its low incidence, there is insufficient clinical awareness and diagnostic experience with primary hepatic malignant vascular tumors (PHMVTs). The aim of our study was to investigate the imaging and clinicopathological features of patients with PHMVTs and analyze the clinicopathological correlations.

**Methods:**

We retrospectively analyzed 42 patients who had pathologically confirmed PHMVT during the period from June 2012 to December 2019 and enrolled them in our study. The computed tomography (CT) and magnetic resonance (MR) images and pathological findings of each patient were recorded.

**Results:**

There were more female (29/42) than male patients. The imaging features of primary hepatic angiosarcoma (PHA) (*n* = 11) included ill-defined margins (11/11, 100%), necrosis (5/11, 45%), calcification (3/11, 27%) and “slow in-slow out” centripetal enhancement (7/11, 64%). Patients with epithelioid hemangioendothelioma (EHE) (*n* = 15) presented with ill-defined margins (15/15, 100%), necrosis (6/15, 40%), calcification (2/15, 13%), “fast in-slow out” centripetal enhancement (10/15, 67%), halo sign (15/15, 100%), pseudocapsule sign (4/15, 27%), lollipop sign (2/15, 13%) and capsule retraction sign (2/15, 13%). Patients with malignant hemangiopericytoma (MHP) (*n* = 3) showed ill-defined margins (3/3, 100%), necrosis (3/3, 100%) and “fast in-slow out” progressive enhancement (3/3, 100%). Infantile hemangioendotheliomas (IHEs) (*n* = 13) were defined by ill-defined margins (7/13, 54%), necrosis (8/13, 62%), calcification (5/13, 38%) and “fast in-slow out” centripetal enhancement (13/13, 100%). Immunohistochemistry showed strong positive expression of CD31, CD34, ERG, FaVIII and FLI-1. Patients with IHE (96 months) and EHE (88 months) had the longest survival times, followed by those with MHP (23 months), while patients with PHA (15 months) had the shortest survival time.

**Conclusion:**

On CT and MR images, most PHMVTs were ill-defined, heterogeneous, hypervascular masses with centripetal progressive enhancement and possibly calcification, especially in female patients. The prognosis of patients with PHMVT was associated with the pathological type of the tumor.

## Background

Primary hepatic malignant vascular tumors (PHMVTs) are a type of tumor that comprises different hepatic vascular components [1]. Benign hemangiomas are more commonly seen clinically, whereas the occurrence of PHMVT is rare. The PHMVT family consists of primary hepatic angiosarcoma (PHA), epithelioid hemangioendothelioma (EHE), malignant hemangiopericytoma (MHP) and infantile hemangioendothelioma (IHE) [2]. The etiology of PHMVT is unclear, and previous literature has reported that the occurrence of PHMVT may be related to oral contraceptive use, progesterone disorders, vinyl chloride contamination, or liver trauma [3]. Due to its low incidence, there is insufficient clinical awareness of and diagnostic experience with PHMVT [4]. A small number of case reports have demonstrated the clinical manifestations of PHMVT [1–5]; however, there are still no imaging characteristic and clinicopathological correlation reports supported by a sufficient amount of data.

The diagnosis of PHMVT mainly depends on histology and multiple vascular endothelial biomarkers. PHMVT is classified according to vimentin, VIIa, HLA-DR and CD34 expression [6]. The literature has reported that the pathological subtype of PHMVT is significantly related to its prognosis [6]. PHA showed the best prognosis, followed by MHP, and IHE exhibited the worst prognosis [7]^.^ Certainly, different pathological types are also associated with different imaging features. Few systematic reports have characterized the imaging features of PHMVT according to pathological type [8–10]. Furthermore, previous case reports and literature reviews have not systematically summarized imaging manifestations and clinical features. In view of this, the aim of this study was to summarize the imaging findings and clinicopathological characteristics of PHMVT and analyze the clinicopathological correlations.

## Methods

This study was approved by the Institutional Review Board of the First Affiliated Hospital of Zhengzhou University. Informed consent was waived due to the retrospective nature of the study.

### Subject enrollment

We retrospectively analyzed 42 patients who had pathologically confirmed PHMVT during the period from June 2012 to December 2019 and enrolled them in our study. Among them, 11 patients with primary hepatic angiosarcoma (PHA), 15 with epithelioid hemangioendothelioma (EHE), 3 with malignant hemangiopericytoma (MHP) and 13 with infantile hemangioendothelioma (IHE) were included in the study for imaging observation and clinical investigation. Pathology findings, demographic features, laboratory results, clinical characteristics, treatment outcomes, and imaging data were recorded.

### Image acquisition

Thirty-eight patients underwent CT (9 with PHA, 14 with EHE, 2 with MHP, 13 with IHE), ten patients underwent MRI (4 with PHA, 4 with EHE, 2 with MHP, 1 with IHE), and seven patients underwent both CT and MRI (2 with PHA, 3 with EHE, 1 with MHP, 1 with IHE).

### CT protocol

Thirty-eight patients underwent CT from the diaphragm to the iliac crest. Unenhanced CT and dual-phase contrast enhancement spectral spiral CT scans were performed using a spectral CT scanner (Discovery CT 750, GE Healthcare, Waukesha, WI, USA). Contrast medium containing 350 mg of iodine per ml (Omnipaque™; GE Healthcare, Cork, Ireland) was injected at a flow rate of 3 ml/s via the elbow vein. The dose of the contrast medium was calculated as 1.5 ml per kg body weight. Scanning was triggered when the CT value of the aortic arch reached 100 HU. Contrast-enhanced CT images were acquired with a scanning delay of 30 s in the arterial phase and 70 s in the portal venous phase after the start of intravenous contrast medium injection [11].

### MRI protocol

Eleven patients underwent magnetic resonance imaging (MRI). MR scanning was performed on a 3.0 T MRI scanner (Discovery MR750 HD, GE Healthcare, Waukesha, WI, USA) using a torso coil. Patients were positioned in the supine position, feet first. The main imaging sequences included the following: 1) three-dimensional double-echo steady state (SPGR-BH-3DDE) axial in-phase and opposed-phase T1-weighted imaging (T1WI); 2) turbo spin echo (TSE) fat-suppression T2-weighted imaging (T2WI); 3) intravoxel incoherent motion (IVIM) biexponential model of diffused weighted imaging (DWI); and 4) liver acquisition with volume acceleration (LAVA) dynamic enhanced imaging. The contrast agent Gd-DTPA (Magnevist, Bayer Schering Pharma, Berlin, Germany) was injected with a dose of 0.1 mmol/kg into the antecubital vein through a pump injector (Medrad, Warrendale, Pennsylvania, USA) at a flow rate of 2.5 mL/s, after which 20 mL of saline was injected at the same rate. The enhanced MRI series included six phases, including two continuous scans each at 12 s and 50 s and one scan each at 90 s and 150 s after contrast agent injection [12].

### Imaging analysis

Two radiologists (Y.Z. and P.H.) with 12 and 10 years of experience in abdominal CT/MRI diagnosis, respectively, independently measured and analyzed the imaging in a blinded and randomized manner at a spectral imaging workstation (Advantage for Windows, version 4.6; GE Healthcare, Waukesha, WI, USA). When there was a discrepancy between interpretations, a consensus was achieved through discussion. The following imaging features of each tumor were assessed: number (single or multiple), site (segment or lobe), size, shape (round or irregular), margin (well-defined or ill-defined), capsule (present or absent), density/intensity (hypo-, iso-, or hyperdense/intense relative to normal liver), cystic/necrotic, calcification, hemorrhage, tumor metastasis, intratumoral blood vessel, enhancement pattern, enhancement degree (hyper- or hypovascular) and typical sign. The attenuation, signal intensity, enhanced pattern and enhanced degree were measured relative to the background liver. The necrotic center and blood vessels were carefully avoided during region of interest (ROI) placement. Measurement of the ROI was repeated three times, and the average of the measurements was used for the final analysis. The basic patient information included patient age, sex, symptoms, treatment duration, pathological results and immunohistochemical findings.

### Pathological test

The pathological results were confirmed through surgery (*n* = 34) and CT/MRI-guided percutaneous biopsy (*n* = 8). The tumor tissue was examined using hematoxylin-eosin (HE) staining and immunohistochemical (IHC) examination. Immunohistochemistry (IHC) analysis of paraffin-embedded sections was performed using the avidin–biotinylated peroxidase complex method. Antibodies used in this study included those against the following: epithelial markers such as cytokeratin (CK), hepatocytes and CK8/18; vascular endothelial markers such as CD31, CD34, ERG, FaVIII and FLI-1; mesenchymal markers such as vimentin, epithelial membrane antigen (EMA), CD117 and Dog-1; and perivascular epithelioid cell markers such as human melanoma black (HMB45) monoclonal antibody and Melan-A. The Ki-67 cell proliferation index (%) was tested accordingly. Pathological images were reviewed independently by two pathologists.

## Results

### Patient characteristics

The eleven patients with PHA included 4 males and 7 females, with a median age of 52 years (range, 39–74 years). There were more female EHE patients than males, and the patient ages ranged from 29 to 69 years (median age: 41 years). Of the patients with MHP and IHE, there were 2 males and 7 females, with median ages of 47 years (range, 32–55 years) and 3 months (0.2–24 months), respectively. The patients with PHA presented with clinical symptoms of upper abdominal pain (*n* = 4), loss of appetite and weight (*n* = 4), fever (*n* = 1), jaundice and diarrhea (*n* = 1), and asymptomatic (*n* = 1). Patients with EHE exhibited upper abdominal pain (*n* = 4), loss of appetite and weight (*n* = 1), fever (*n* = 1), and asymptomatic patients (*n* = 3). Of the two patients with MHP, they presented with abdominal pain (*n* = 1) or were asymptomatic (*n* = 1). The patients with IHE developed fever (*n* = 2), jaundice and diarrhea (*n* = 3), delayed growth (*n* = 1) and thrombocytopenia (*n* = 1). The remaining six patients with IHE presented an abdominal mass, which was found by their parents. While three patients with PHA were positive for hepatitis B virus antibodies or cirrhosis, two patients had coronary heart disease, four patients had hypertension, and three patients had diabetes at the time of administration. For patients with EHE, one patient each was positive for hepatitis B virus antibodies or cirrhosis and coronary heart disease, while hypertension and diabetes were present in three patients each. The basic characteristics of the patients with PHMVT are described in Table 1.

**Table 1:** Basic characteristic of the patients with primary hepatic malignant vascular tumor

Variables	PHA	EHE	MHP	IHE
**No. of patients**	11	15	3	13
**Gender**				
Male	4 (36%)	2 (13%)	1 (33%)	6 (46%)
Female	7 (64%)	13 (87%)	2 (67%)	7 (54%)
**Age, median, y (range)**	52 (39–74) y	41 (29–69) y	47 (32–55)y	3 (0.2–24)m
**Clinical symptom**				
Upper abdominal pain	4 (36%)	4 (27%)	2 (67%)	0
Loss of appetite and weight	4 (36%)	1 (7%)	0	0
Fever	1 (9%)	1 (7%)	0	2 (15%)
Jaundice and diarrhea	1 (9%)	0	0	3 (23%)
Growth retardation	0	0	0	1 (8%)
Thrombocytopenia	0	0	0	1 (8%)
Abdominal mass	0	0	0	6 (46%)
Asymptomatic patient	1 (9%)	3 (20%)	1 (33%)	0
**Medical history**				
Hepatitis-B/ Cirrhosis	3 (27%)	1 (7%)	0	0
CHD	2 (18%)	1 (7%)	0	0
Hypertension	4 (36%)	3 (20%)	0	0
Diabetes	3 (27%)	3 (20%)	0	0
**Treatment strategy**				
Hepatectomy	5 (45%)	15 (100%)	2 (67%)	13 (100%)
TACE	1 (9%)	0	0	0
Radiofrequency ablation	1 (9%)	0	0	0
Chemotherapy	2 (18%)	0	0	0
Antiangiogenic therapy	1 (9%)	0	0	0
Postoperative chemotherapy	1 (9%)	0	1 (33%)	0
**Median survival time, months**	15	88	23	96

Note: *PHA* Primary hepatic angiosarcoma; *EHE* Epithelioid hemangioendothelioma; *MHP* Malignant hemangiopericytoma; *IHE* Infantile hemangioendothelioma; *CHD* Coronary heart disease; *TACE* Transarterial chemoembolization

### Pathological features

**Fig. 1:**
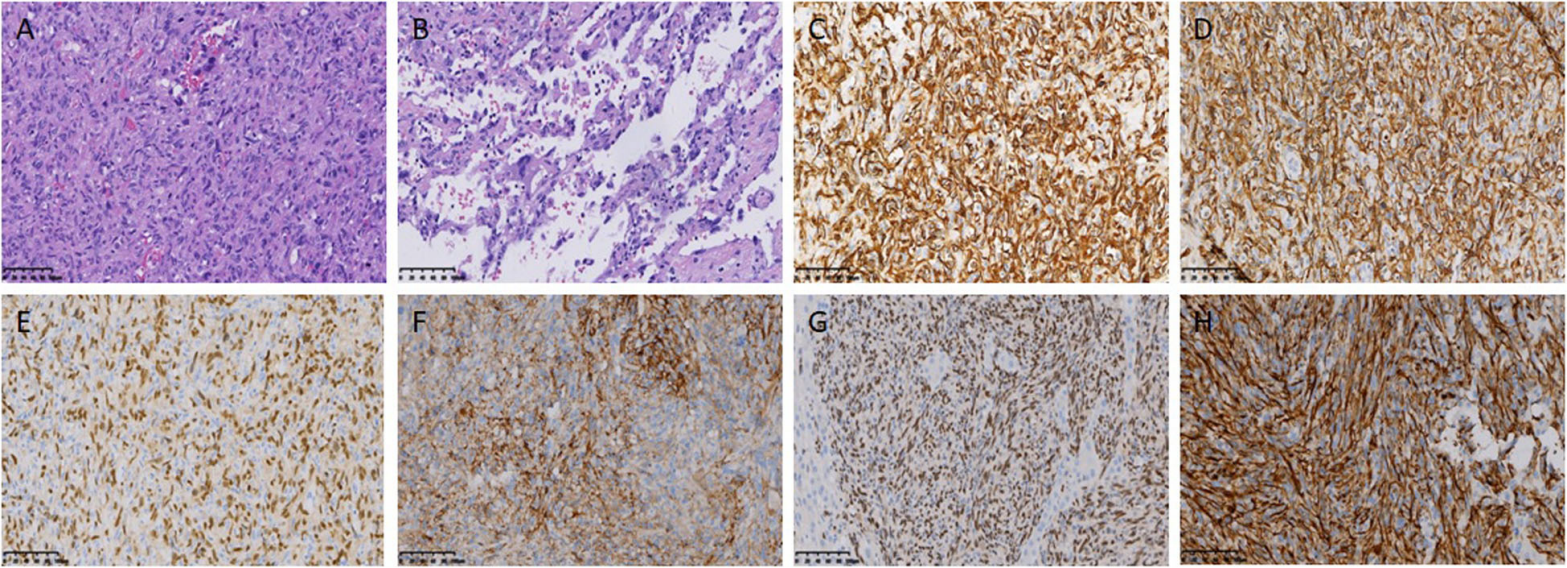
Histological examination of a 75-year-old female patient with PHA. H&E-stained images demonstrate that the tumor cells are pleomorphic overlying along the blood sinuses (× 200) (**a**,**b**); immunohistochemistry demonstrates that the cells are positive for CD31 (**c)**, CD34 (**d**), ERG (**e**), FaVIII (**f**) FLI-1 (**g**) and vimentin (**h**) (× 100)

**Fig. 2:**
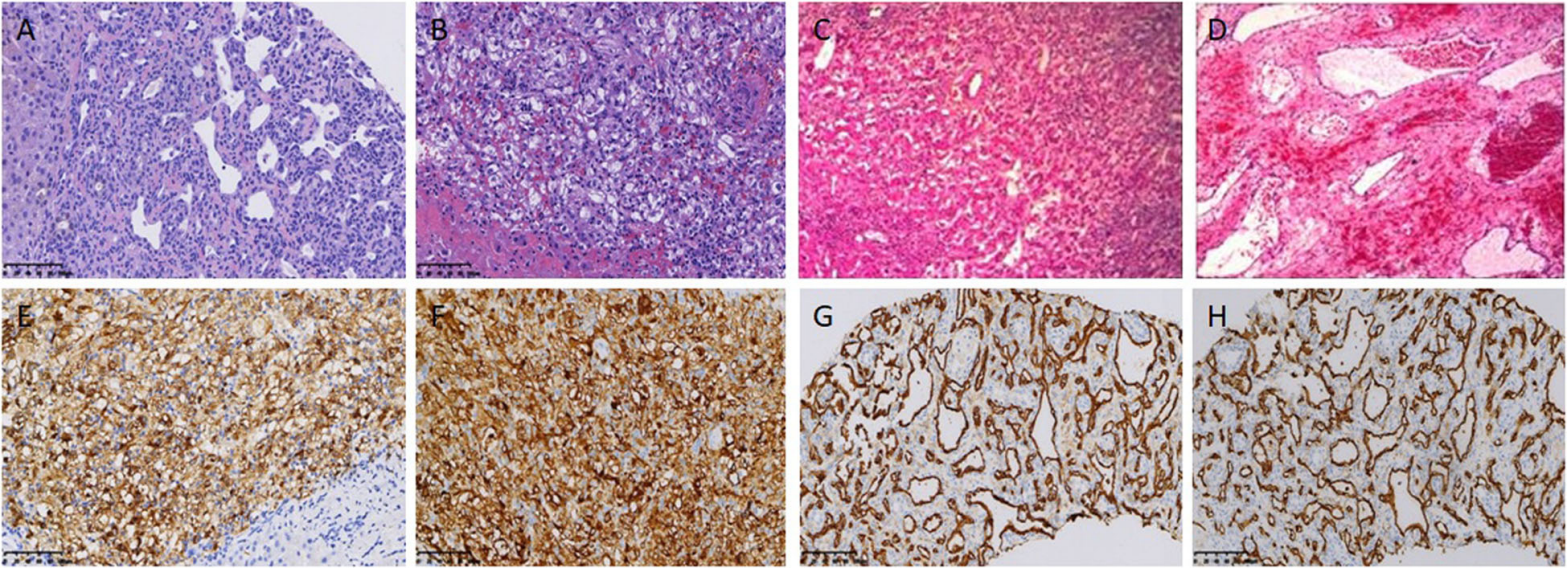
Histological examination with H&E-stained images of EHE demonstrates vascular differentiated dendritic cells and epithelioid cells of the intracellular vascular lumen (× 200) (**a**), MHP exhibits vascular epidermal cells radiating around the capillary outer membrane (× 200) (**b**), and IHE shows papillary hyperplasia in vascular endothelial cells (× 200) (**c**), (× 400) (**d**); immunohistochemistry demonstrates that the cells of MHP were positive for HMB45 (**e**), Melan-A (**f**), CD31 (**g**) and CD34 (**h**) (× 100)

The sectioned surface of a PHA shows dark-red tissue with necrosis, hemorrhage and calcification. EHE shows a tough gray-white or brown tissue with calcification, and MHP displays a light-brown tissue with degeneration and hemorrhage. A section of IHE reveals a red-brown tissue with capillaries. Most tissues had unclear boundaries between the tumor edge and normal tissue. Histological examination demonstrated that PHA consists of neoplastic endothelial cells of different sizes, forming a cavernous vascular lumen-like structure, and some tumor cells form a papillary sinusoidal or even a solid structure. The tumor cells are spindle-shaped, large in size, with obvious nuclear atypia and multinucleated giant cells (Fig. 1a,b); EHE had vascular differentiated dendritic cells or epithelioid cells of the intracellular vascular lumen (Fig. 2a); MHP exhibited vascular epidermal cells radiating around the capillary outer membrane (Fig. 2b); and IHE showed papillary hyperplasia in vascular endothelial cells, which protruded into the lumen in clusters (Fig. 2c,d). As summarized in Table 2, IHC showed strong positive expression of the vascular endothelial markers CD31, CD34, ERG, FaVIII and FLI-1 in PHMVT. PHA, EHE and IHE showed partial positive expression of CK, hepatocytes and CK8/18 antibodies for the epithelial component (Fig. 1c-g, Fig. 2g, h). Vimentin, EMA and CD117 were expressed in a portion of the tested PHAs and EHEs (Fig. 1h). HMB45 and Melan-A, typical perivascular epithelioid cell markers, were positively expressed in MHP (Fig. 2e, f).

**Table 2:** Immunohistochemical biomarkers in primary hepatic malignant vascular tumor

Immunohistochemical biomarkers	PHA	EHE	MHP	IHE
**Epithelial marker**				
CK	7 (64%)	8 (53%)	0	2 (15%)
Hepatocytes	6 (55%)	4 (27%)	0	0
CK8/18	6 (55%)	6 (40%)	0	0
**Vascular endothelial marker**				
CD31	11 (100%)	15 (100%)	3 (100%)	13 (100%)
CD34	11 (100%)	15 (100%)	3 (100%)	13 (100%)
ERG	9 (82%)	13 (87%)	2 (67%)	13 (100%)
FaVIII	9 (82%)	9 (60%)	1 (33%)	7 (54%)
FLI-1	9 (82%)	9 (60%)	1 (33%)	7 (54%)
**Mesenchymal marker**				
Vimentin	4 (36%)	2 (13%)	0	0
EMA	3 (27%)	0	0	0
CD117	2 (18%)	0	0	0
Dog-1	0	0	0	0
**Perivascular epithelioid cell marker**				
HMB45	0	0	2 (67%)	0
Melan-A	0	0	2 (67%)	0
**Cell proliferation marker**				
Ki-67, median, % (range)	30 (1–60)	5 (2–20)	30 (10–30)	10 (1–20)

Note: *PHA* Primary hepatic angiosarcoma; *EHE* Epithelioid hemangioendothelioma; *MHP* Malignant hemangiopericytoma; *IHE* Infantile hemangioendothelioma; *CK* Cytokeratin; *EMA* Epithelial membrane antigen; *CD* Cluster of differentiation; *HMB45* Human melanoma black monoclonal antibody

### Follow-up data

Thirty-five patients (5 with PHA, 15 with EHE, 2 with MHP, and 13 with IHE) underwent surgical resection, and the remaining patients with PHA underwent transarterial chemoembolization (TACE) (*n* = 1), radiofrequency ablation (*n* = 1), chemotherapy (*n* = 2), antiangiogenic therapy (*n* = 1) and postoperative chemotherapy (*n* = 1). One patients with MHP underwent postoperative chemotherapy. All patients were followed up until September 2019. Patients with IHE (96 months) and EHE (88 months) had the longest median survival times, followed by MHP (23 months), and patients with PHA (15 months) had the shortest survival time.

### Imaging findings

As described in Tables 3, 9 of 11 (82%) PHAs, 15 of 15 (100%) EHEs and 2 of 3 (67%) MHPs involved multiple liver segments, while 8 of 13 (62%) were solitary lesions of the liver. The mean tumor diameters for the solitary lesions were 6.3 cm (range, 1.1–8.6 cm) for PHA, 4.7 cm (range, 1.5–9 cm) for EHE, 9.8 cm (range, 7.2–10.2 cm) for MHP, and 24.44 cm (range, 1–6.6 cm) for IHE. Twenty-seven patients (9/11 with PHA, 10/15 with EHE, 1/3 with MHP, 7/13 with IHE) had irregular lesions. Most tumors (36/42) had ill-defined margins, and 40/42 had no capsules. Tumor cysts/necrosis, characterized by a nonenhanced cystic appearance, was present in 21 patients (5/11 with PHA, 6/15 with EHE, 3/3 with MHP, 8/13 with IHE). Hemorrhage presented as high density on nonenhanced CT or hypersignal intensity on T1W images in 1 patient with PHA, and calcification was identified in 10 patients (3/11 with PHA, 2/15 with EHE, 5/13 with IHE) in the form of punctate foci on CT. Dysmorphic vessels were identified in 45% (19/42) of tumors (4/11 PHA, 10/15 EHE, 5/13 IHE) by contrast-enhanced CT or MRI.

**Table 3:** Image characteristics of primary hepatic malignant vascular tumor

Image findings	PHA	EHE	MHP	IHE
**Liver involvement**				
Single	2 (18%)	0	1 (33%)	8 (62%)
Multiple	9 (82%)	15 (100%)	2 (67%)	5 (38%)
**Number of segments involved**				
1	3 (27%)	1 (7%)	0	7 (54%)
2 ~ 4	7 (64%)	5 (33%)	2 (67%)	4 (31%)
> 5	1 (9%)	9 (60%)	1 (33%)	2 (15%)
**Tumor shape**				
Round	2 (18%)	5 (33%)	2 (67%)	6 (46%)
Irregular	9 (82%)	10 (67%)	1 (33%)	7 (54%)
**Margin**				
Well-defined	0	0	0	6 (46%)
Ill-defined	11 (100%)	15 (100%)	3 (100%)	7 (54%)
**Capsule**				
Present	2 (18%)	0	0	0
Absent	9 (82%)	15 (100%)	3 (100%)	13 (100%)
**Density**				
Hypo-	11 (100%)	12 (80%)	2 (100%)	13 (100%)
Iso-	0	3 (20%)	0	0
Hyper-	0	0	0	0
**T1WI**				
Hypo-	3 (75%)	4 (100%)	2 (100%)	1 (100%)
Iso-	0	0	0	0
Hyper-	1 (25%)	0	0	0
**Fat-suppressed T2WI**				
Hypo-	0	0	0	0
Iso-	0	0	0	0
Hyper-	4 (100%)	4 (100%)	2 (100%)	1 (100%)
**Diffused weighted imaging (DWI)**				
Hypo-	1 (25%)	0	0	0
Iso-	0	0	0	0
Hyper-	3 (75%)	4 (100%)	2 (100%)	1 (100%)
**Cystic/necrosis**				
Present	5 (45%)	6 (40%)	3 (100%)	8 (62%)
Absent	6 (55%)	9 (60%)	0	5 (38%)
**Calcification**				
Present	3 (27%)	2 (13%)	0	5 (38%)
Absent	8 (73%)	13 (87%)	3 (100%)	8 (62%)
**Hemorrhage**				
Present	1 (9%)	0	0	0
Absent	10 (91%)	15 (100%)	3 (100%)	13 (100%)
**Morphological type**				
Nodules type (d < 5 cm)	2 (18%)	0	2 (67%)	5 (38%)
Mass type (d ≥ 5 cm)	7 (65%)	0	1 (33%)	3 (24%)
Diffused micronodules type	2 (18%)	15 (100%)	0	5 (38%)
**Enhanced pattern**				
“Slow in-slow out” centripetal enhancement	7 (64%)	5 (33%)	0	0
“Fast in-slow out” centripetal enhancement	4 (36%)	10 (67%)	3 (100%)	13 (100%)
**Enhanced degree**				
Hyper-vascular	8 (73%)	9 (60%)	2 (67%)	13 (100%)
Hypo-vascular	3 (27%)	6 (40%)	1 (33%)	0
**Metastases**				
Lung	2 (18%)	7 (47%)	1 (33%)	0
Spleen	1 (9%)	0	0	0
Bone	1 (9%)	0	1 (33%)	0
Retroperitoneal space	3 (27%)	1 (7%)	0	0
**Intratumoral blood vessel**	4 (37%)	10 (67%)	0	5 (38%)
**Typical sign**				
“Psuedocapsule sign”	0	4 (27%)	0	0
“Halo sign”	2 (18%)	15 (100%)	3 (100%)	0
“Lollipop sign”	0	2 (13%)	0	0
“Capsule retraction sign”	0	2 (13%)	0	0

Note: *PHA* Primary hepatic angiosarcoma; *EHE* Epithelioid hemangioendothelioma; *MHP* Malignant hemangiopericytoma; *IHE* Infantile hemangioendothelioma

**Fig. 3:**
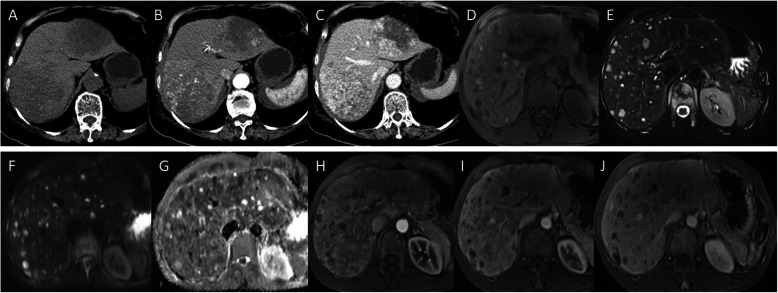
Typical cases of PHA. A 75-year-old female patient with PHA. The unenhanced CT image shows an irregular, ill-defined hypoattenuated hepatic mass (**a**), marginal patchy enhancement on the arterial phase (**b**), and progressive, gradual filling enhancement on the portal venous phase (**c**). A 43-year-old male patient with PHA. The MR images show hypo/hyperintense diffuse lesions on T1WI (**d**), hyperintense signals on T2WI (E), hypo/hyperintense signals on DWI (**f**) and the apparent diffusion coefficient (ADC) map (**g**), marginal patchy enhancement on the arterial phase (**h**), and progressive, gradual filling enhancement on the portal venous phase (**i**) and delayed phase (**j**)

**Fig. 4:**
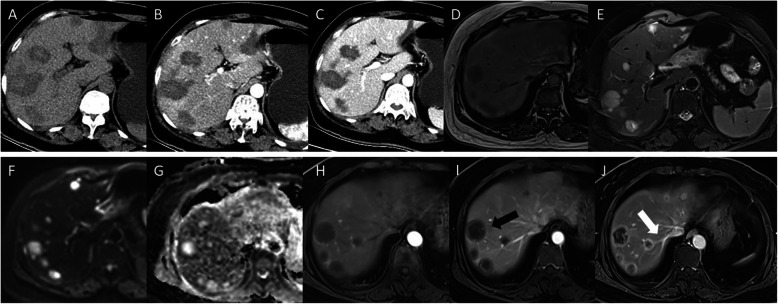
A 52-year-old woman with EHE. The nonenhanced CT scan (**a**) reveals multiple ill-defined homogeneous hypoattenuated lesions. The lesions demonstrate marginal enhancement in the arterial phase (**b**) and heterogeneous progressive centripetal enhancement in the portal vein (**c**). The MRI scan (**d**, T1WI in-phase; **e**, fat-suppressed T2WI; **f**, DWI; **g**, ADC map) shows hypointensity on T1WI, hyperintensity on T2WI and high signal intensity on DWI and the ADC map. The enhancement pattern on dynamic contrast-enhanced MRI (**h**, arterial phase; **i**, portal venous phase; **j**, delayed phase) corresponds with that on CT. The lesion presents with the halo sign (black arrow) and lollipop sign (white arrow)

**Fig. 5:**
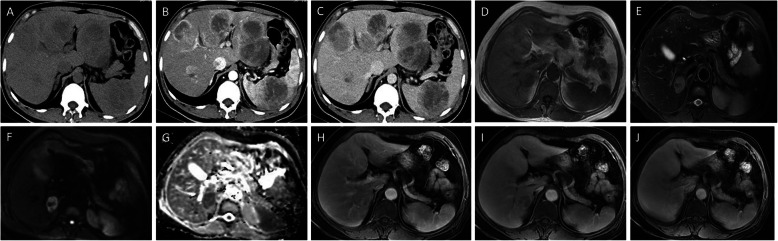
Typical cases of MHP. A 31-year-old male patient. The unenhanced CT image shows multiple hypodense hepatic lesions and spleen metastasis with ill-defined margins (**a**), heterogeneous marginal enhancement on the arterial phase (**b**), and progressive centripetal enhancement without full filling on the portal venous phase (**c**). A 55-year-old female patient with PHA. The MR images show hypointense lesions at the right hepatic lobe on T1WI (**d**), hyperintensity on T2WI (**e**), hyperintensity on DWI (F), hypointensity on the ADC map (**g**), slightly marginal enhancement on the arterial phase (H), patchy filling enhancement on the portal venous phase (**i**) and progressive, gradual filling enhancement on the delayed phase (**j**)

**Fig. 6:**
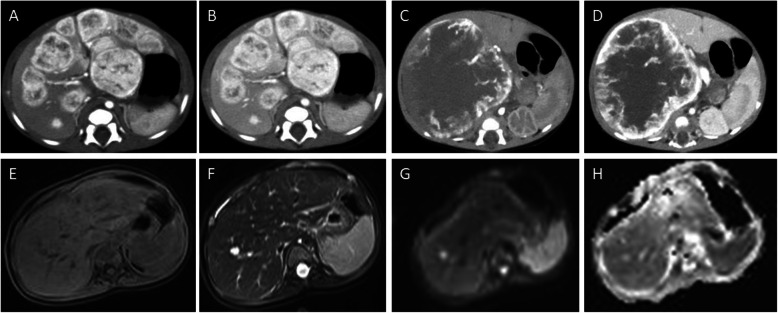
Typical cases of IHE. A 2-month-old female patient with IHE. The enhanced CT images display multiple hepatic lesions with markedly heterogeneous enhancement (**a**), gradually filling from the periphery to the center (**b**). A 2-month-old male patient with IHE. The enhanced CT images show a very large hepatic lesion with markedly marginal enhancement with nourishing blood vessels (**c**), gradually filling from the periphery to the center (**d**). A 7-month-old female patient with IHE. The MR images show hypointense lesions in the right hepatic lobe on T1WI (**e**), hyperintensity on T2WI (**f**), and hyperintensities on DWI (**g**) and the ADC map (**h**)

On unenhanced images, most PHMVTs presented with hypodense or hypo/hyperintense signals on T1WI/fat-suppressed T2WI. One PHA with central hemorrhage showed hyperintense signals on T1WI images. The enhancement patterns of the tumors varied depending on the morphological characteristics. Sixty-four percent (7/11) of PHAs were characterized by central and marginal patchy enhancement, and the degree of enhancement was higher than that of the surrounding normal liver parenchyma in the arterial phase. The delayed phase showed progressive, gradual filling enhancement (Fig. 3). EHE was enhanced in 67% (10/15) of tumors in the arterial phase with ring-like or marginal enhanced regions, and the portal venous and delayed phases showed progressive centripetal enhancement. All EHEs presented with the halo sign, while the pseudocapsule sign, lollipop sign and capsule retraction sign were present in a portion of the EHEs (Fig. 4). MHPs showed heterogeneous enhancement in the arterial phase and progressive centripetal enhancement without full filling (Fig. 5). IHEs displayed markedly heterogeneous reinforcement, gradually filling from the periphery to the center (Fig. 6). Metastasis was identified in 7 patients with PHA (2 in the lung, 1 in the spleen, 1 in bone, and 3 in the retroperitoneal space), 8 patients with EHE (7 in the lung and 1 in the retroperitoneal space) and 2 patients with MHP in the lung and bone (Table 3).

## Discussion

The incidence of PHMVT is relatively low among primary hepatic tumors. The typical clinical symptoms are abdominal pain and loss of appetite and weight [2]. Liver function and tumor biomarkers are usually normal. Positive CD31, CD34 and VII are exhibited in immunohistochemical tests [5]. PHA, EHE and MHP tend to occur in middle-aged females, whereas IHE mostly occurs in infants younger than 6 months of age. PHMVT is prone to pulmonary metastasis and is easily misdiagnosed clinically [6]. To date, only a few cases of PHMVT have been described worldwide [1–10]. Thus, we designed the present study to explore the CT manifestations of PHMVT to improve the understanding of this disease.

PHA is a highly malignant stromal tumor originating from hepatic sinus vascular endothelial cells, accounting for 2% of all primary hepatic tumors [13]. Recent studies have reported that the incidence of PHA may be associated with liver fibrosis and cirrhosis [14]. Three PHA cases were HBsAg positive in our study. Seven cases with a mass showed a rich blood supply, which was characterized as nodular or patchy enhancement, and the degree of enhancement was slightly higher than that of the liver parenchyma. The portal venous and delayed phases showed progressive enhancement. Due to hemorrhage and necrotic or fibrous components, the parenchyma filling was slow, and the lesion could not be fully filled. However, two cases presented with diffuse nodules in our study and had a relative lack of blood supply. The enlargement of the enhancement area was “centripetal” or “centrifugal”. We found that the PHAs had abundant blood sinuses, and nourishing blood vessels could be seen after enhancement, which represents the invasion of blood vessels.

EHE is an angiogenic tumor with relatively low malignant potential and morphological characteristics between those of hemangioma and angiosarcoma [15]. In this study, all EHEs presented as multiple fused nodules under the hepatic capsule, with or without calcification. The halo sign, capsule contraction sign and lollipop sign are the typical manifestations of EHEs. In this study, all 15 patients presented with a halo sign, that is, small hemorrhagic foci inside the lesion with hyperintense and hypointense signals of the edema zone around the lesion. The capsule contraction sign is mainly related to the abundant fibrous components of the tumor [16]. Hepatic capsule retraction is caused by the fibrous matrix withdrawal symptom pulling the adjacent liver capsule [16]. Owing to hepatic sinus and portal venous invasion, the lollipop sign may be a manifestation of vascular tumor invasion [17]. In the lollipop sign, the lesion around the vein branch is similar to the “candy”, while the branch of the hepatic or portal vein is similar to the “stick” [17]. In our study, most EHEs demonstrated heterogeneous persistent or progressive enhancement. The reason may be that the contrast agent gradually infiltrated into the fibrous tissue inside the tumor and developed over time, manifesting as centripetal enhancement.

MHP is a vasogenic malignant tumor that occurs in Zimmermann’s pericytes or vascular peripheral multifunctional stromal cells [18]. It mainly occurs in the skull, trunk, upper limbs, retroperitoneum, pelvis and lower limbs, and hepatic MHP is rare [18]. The imaging manifestation of MHP is described as a large solitary solid or cystic-solid irregular mass with uneven density, unclear boundaries, hemorrhage and necrosis. Studies have shown that MHPs can be enhanced in a variety of ways from the early to late phase [19]. In our study, 2 patients showed obvious heterogeneous enhancement in the arterial phase, which gradually decreased in the portal venous and delayed phases, with no obvious expansion in the enhancement range. In our study, MHP presented mixed hyper/hypointense signals in both T1WI and T2WI, suggesting hemorrhage and necrosis in the tumor. Thus, the imaging features of MHP are not distinctive, and a diagnosis can be confirmed only with pathology and relative IHC markers.

IHC, originating from the hepatic mesenchymal tissue, is a rare infantile vasogenic tumor [20]. IHCs are mainly divided into two types: type I is a benign lesion that is common in the clinic, and type II is relatively rare with papillary hyperplasia in vascular endothelial cells and has malignant and metastatic potential [20]. In our study, most IHCs showed marginal ring enhancement on the arterial phase and “fast in-slow out” centripetal enhancement, which was basically consistent with the literature. We also found that the high speed of blood flow in the narrow vascular lumen may lead to homogeneous enhancement for small tumors in the arterial phase. However, for tumors with large diameters, the lumen of the blood vessel is wider and irregular, with a disorderly distribution. We believe that these tumors may exhibit a pattern of slow filling and heterogeneous enhancement at different points because of the slow blood flow.

PHMVT is rare in clinical practice. The solid components are significantly enhanced in the arterial phase. EHE, MHP and IHE present with heterogeneous and persistent enhancement without filling, while PHA shows persistent and complete filling in the portal venous phase [8–10]. Imaging findings should be differentiated from hepatic hemangioma, hepatocellular carcinoma and hepatoblastoma [13–20]. 1) Hepatic hemangiomas are common in adults. Hemorrhage and central necrosis are rare. Contrast-enhanced images typically show an enhancement pattern of “fast in-slow out”. 2) Patients with hepatocellular carcinoma often have a history of hepatitis B and cirrhosis, accompanied by increased AFP. Contrast-enhanced images show a “fast in-fast out” enhancement pattern. 3) Hepatoblastoma presents as an asymptomatic solitary mass, affecting more male than female children, followed by increased AFP. The contrast enhancement degree of hepatoblastoma is significantly lower than that of IHE in the arterial phase.

Several limitations in this study deserve consideration. First, a relatively small sample size was included, and more patients need to be enrolled in future studies. Moreover, imaging features obtained with other modalities, such as ultrasound and PET, need to be summarized in further studies. Finally, follow-up data were incomplete due to the retrospective nature of the study, and tumors that were not followed up were not evaluated.

## Conclusion

Most PHMVTs are ill-defined, heterogeneous, hypervascular masses with centripetal progressive enhancement with or without calcification on CT and MR images and are found especially in female patients. The prognosis of patients with PHMVT is associated with the pathological type of the tumor.

## Data Availability

The datasets used and/or analyzed during the current study are available from the corresponding author on reasonable request.

## Abbreviations

PHMVT: Primary hepatic malignant vascular tumors
PHA: Primary hepatic angiosarcoma
EHE: Epithelioid hemangioendothelioma
MHP: Malignant hemangiopericytoma
IHE: Infantile hemangioendothelioma
ck: Cytokeratin
EMA: Epithelial membrane antigen
CD: Cluster of differentiation
HMB45: Human melanoma black monoclonal antibody
